# Organic amendment treatments for antimicrobial resistance and mobile element genes risk reduction in soil-crop systems

**DOI:** 10.1038/s41598-023-27840-9

**Published:** 2023-01-17

**Authors:** Leire Jauregi, Aitor González, Carlos Garbisu, Lur Epelde

**Affiliations:** NEIKER – Basque Institute of Agricultural Research and Development, Basque Research and Technology Alliance (BRTA), Parque Científico y Tecnológico de Bizkaia, P812, 48160 Derio, Spain

**Keywords:** Antimicrobial resistance, Agroecology

## Abstract

Agricultural fertilization with organic amendments of animal origin often leads to antibiotic resistance dissemination. In this study, we evaluated the effect of different treatments (anaerobic digestion, biochar application, ozonation, zerovalent iron nanoparticle application, and spent mushroom substrate addition) on the resistome in dairy cow manure-derived amendments (slurry, manure, and compost). Anaerobic digestion and biochar application resulted in the highest reduction in antibiotic resistance gene (ARG) and mobile genetic element (MGE) gene abundance. These two treatments were applied to cow manure compost, which was then used to fertilize the soil for lettuce growth. After crop harvest, ARG and MGE gene absolute and relative abundances in the soil and lettuce samples were determined by droplet digital PCR and high-throughput qPCR, respectively. Prokaryotic diversity in cow manure-amended soils was determined using 16S rRNA metabarcoding. Compared to untreated compost, anaerobic digestion led to a 38% and 83% reduction in *sul2* and *intl1* absolute abundances in the soil, respectively, while biochar led to a 60% reduction in *intl1* absolute abundance. No differences in lettuce gene abundances were observed among treatments. We conclude that amendment treatments can minimize the risk of antibiotic resistance in agroecosystems.

## Introduction

Antibiotic resistance is an ancient phenomenon^1^; however, anthropogenic activities have increased the prevalence of antimicrobial-resistant microorganisms^2,3^, causing serious human health problems. The transfer of animal and environmental resistomes to humans is a matter of great concern for policymakers. Consequently, in 2006, the European Union banned the use of antibiotics for animal growth promotion^4^. Moreover, the EU Farm to Fork Strategy aims to reduce the overall EU sales of antimicrobials for farmed animals and aquaculture by 50% by 2030^5^.

Although these regulations control the excessive use of antibiotics in livestock farming, none are related to the management and/or treatment of organic amendments of animal origin. Organic amendments of animal origin, which are commonly used for agricultural fertilization, are a source of antibiotic residues because a considerable percentage (30–90%) of antibiotics administered to livestock are discharged in the urine and feces^6^. The application of these organic amendments to soil as organic fertilizers can lead to the emergence and dissemination of antibiotic-resistant bacteria (ARB) harboring antibiotic resistance genes (ARGs) in soils and crops^7^. The principal mechanism underlying the spread of antibiotic resistance is horizontal gene transfer (HGT) via various mobile genetic elements (MGEs), such as conjugative plasmids, integrative conjugative elements, integrons, and transposons^8^.

The many benefits of organic amendments to agricultural soils and crops are undeniable^9^. Therefore, there is an urgent need to develop and implement management practices and treatments to reduce the abundance of ARGs and MGEs in animal-based organic amendments. Possible strategies for achieving this include sanitizing the amendments under certain environmental conditions (e.g., temperature, oxygenation) or with other substances, as well as seeking to immobilize ARB and ARGs by adding agents with a very high surface-to-volume ratio. Anaerobic digestion is the process of microbial decomposition of organic matter in the absence of oxygen^10^. Traditionally, it has been used to reduce the volume of solids from wastewater treatment, producing biogas and other organic compounds^11^. Nevertheless, its use to treat livestock manure is increasing; this process can reduce antibiotic residues, ARB, and ARGs in manure^10,12^. In addition, ozone disinfection destroys bacterial cell membranes^13^ and is widely used in wastewater treatment plants and the food industry. This disinfection process can also remove ARGs^14^.

On the other hand, biochar is a carbon rich material with a large surface area-to-volume ratio generated from the pyrolysis of biomass in the absence of oxygen^15^. It is used as a soil amendment to increase soil carbon sequestration, enhance soil fertility and productivity, reduce the bioavailability of organic compounds^16^, and remove antibiotic residues ^17^. Moreover, nanoscale zerovalent iron particles (nZVI) show high reactivity towards a broad range of pollutants and have a large surface-area-to-volume ratio^18^. For example, they are used for the removal of polycyclic aromatic hydrocarbons and metal(oid)s^19^, as well as bacteria and viruses^20,21^. They are mainly applied in groundwater remediation and, to a lesser extent, soil remediation^22^. Finally, five kilograms of spent mushroom substrate (SMS) are generated in the production of one kilogram of fresh mushrooms [for example, Spain produces around 166 thousand tons of mushrooms per year^23^]. Spent mushroom substrate is therefore generated in large quantities, and for many years, it has accumulated in landfills and has become an environmental problem. SMS has been used in the production of compost^24^ and animal feed^25^, as well as for enzyme extraction^26^, bioremediation^27^, and removal of antibiotic residues by combining their adsorption and biodegradation mechanisms^28^.

In the present study, we evaluated the potential of 11 organic amendment management processes to reduce the abundance of ARGs and MGE genes; the amendments used were cow slurry, manure, and compost. Subsequently, the most promising management strategies were applied to a compost that was used to fertilize soil and grow lettuce plants in a microcosm experiment. Thus, we quantified ARGs and MGE genes in (i) amendments, (ii) amended soils, and (iii) lettuce plants. In addition, we studied the structural diversity of soil bacterial communities. We hypothesized that both absolute and relative abundances of ARGs and MGE genes would be lower in the treated amendments than in the untreated controls, and consequently be lower in the amended soils and lettuce plants grown in these soils.

## Results

### Preliminary study with the three amendments

**Figure 1:**
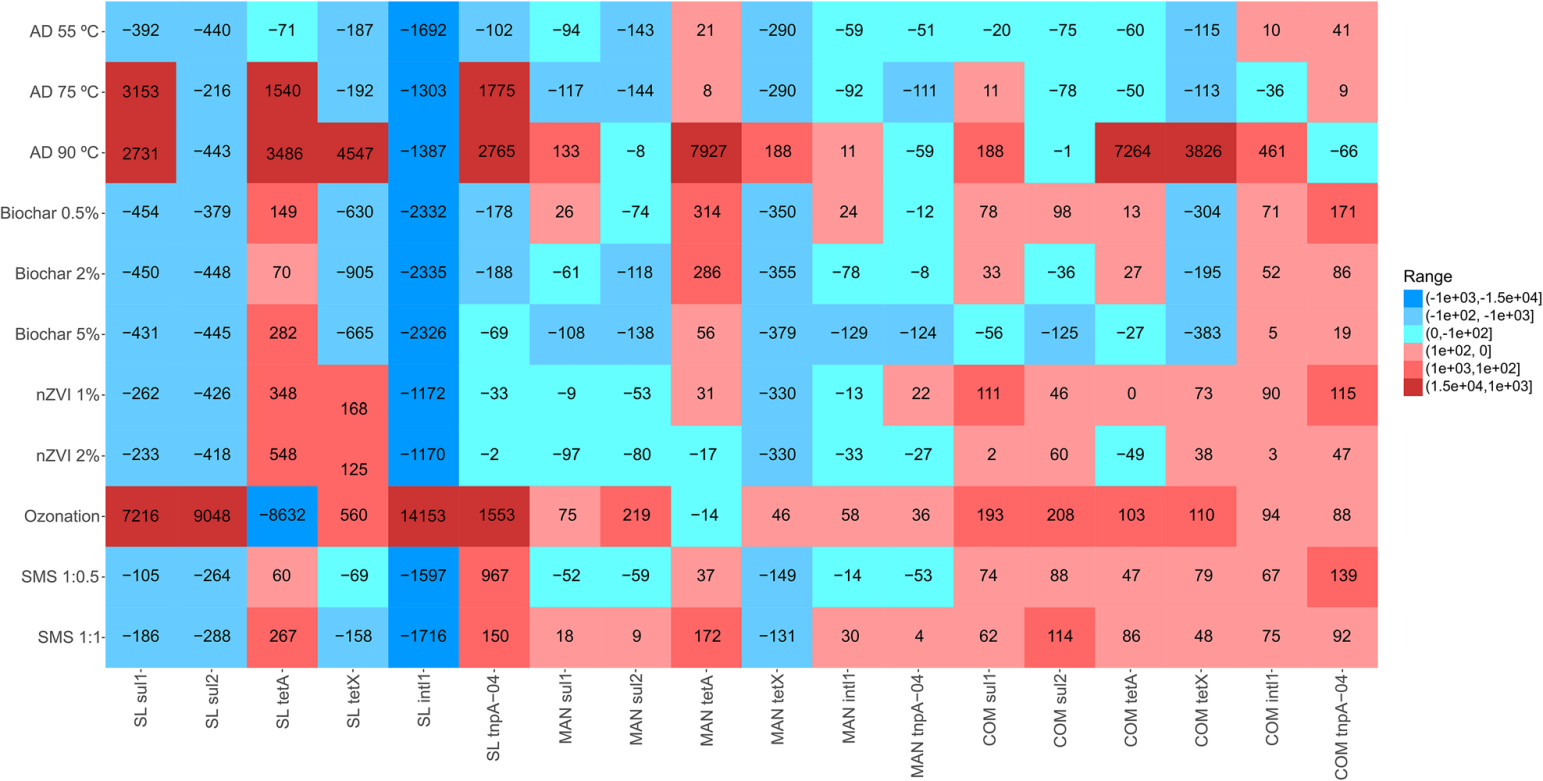
Removal rates of antibiotic resistance genes (ARGs) and mobile genetic element (MGE) genes under different treatments in three organic amendments (n = 1). Negative values indicate a reduction and positive values an increase in absolute gene abundances, compared to its respective control treatment. Treatments: AD: anaerobic digestion; nZVI: zerovalent iron nanoparticles; SMS: spent mushroom substrate. Amendments: SL: slurry; MAN: manure; COM: compost.

Eleven management processes were tested in a preliminary experiment, performed with slurry, manure and compost, that lasted 21 days. The ARG and MGE gene removal rates in each treatment for every amendment were evaluated using a droplet digital PCR (ddPCR; Fig. 1). Negative values indicated that the management process was successful in gene elimination; conversely, positive values indicated that compared to that in the control treatment, gene abundance increased from day 0 to day 21 in the studied treatment. To determine the success of the management processes in terms of the removal rate of the studied genes, we investigated both the number of genes reduced and the magnitude of their removal.

In slurry, the anaerobic digestion (AD) 55 °C treatment showed the greatest success in removing all tested genes; the following most successful treatments were the biochar treatments for all genes except *tetA*. On the other hand, the ozonation treatment presented the opposite trend, showing a significant increase in all genes except *tetA*.

Regarding the manure, the nZVI 2% treatment had the greatest success in removing all tested genes, followed by the AD 55 °C, AD 75 °C, biochar 2%, biochar 5%, and SMS 1:0.5 treatments, which failed only with the *tetA* gene. In contrast, the ozonation and SMS 1:1 treatments increased the removal rates of five out of the six genes.

In compost, the AD 55 °C, AD 75 °C, and biochar 5% treatments showed the greatest success in removing ARGs and MGE genes. On the other hand, the nZVI 1%, ozonation, SMS 1:0.5, and SMS 1:1 treatments showed the opposite trend, increasing the removal rate of every gene.

### Soil and lettuce antimicrobial resistance and mobile element genes

Of the three investigated amendments, we selected compost for the microcosm experiment because it is commonly used to fertilize lettuce. The compost management treatments AD 75 °C and biochar 5% were chosen because they were the most promising treatments in the preliminary study. In an additional treatment, a 5% biochar addition to the soil was tested in order to compare the application of biochar to the amendment with its direct application to the soil.

**Figure 2:**
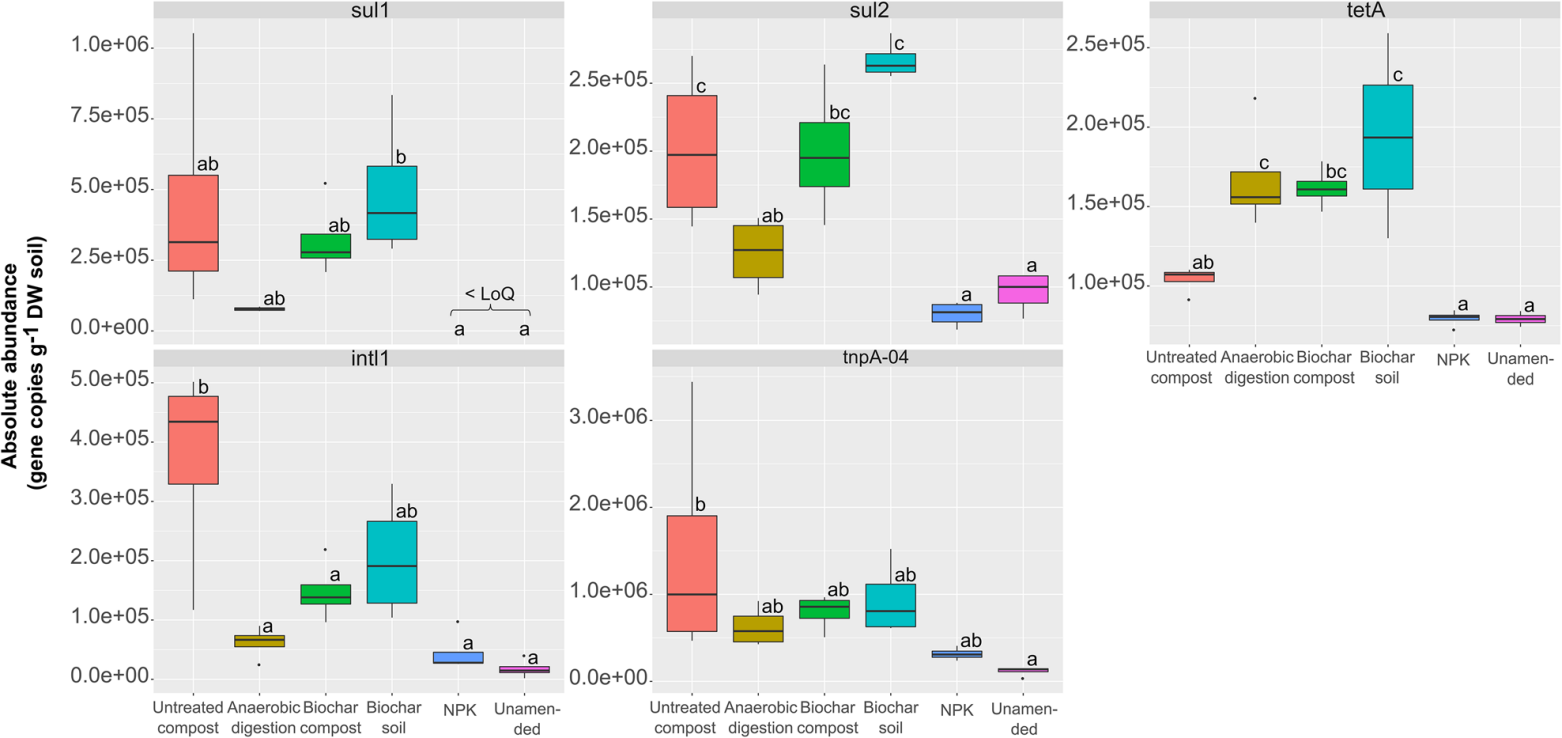
Absolute abundances of antibiotic resistance genes (ARGs) and mobile genetic element (MGE) genes in the soil samples according to the ddPCR analysis, expressed as gene copies per g of DW soil (n = 4). Different letters indicate statistically significant differences among treatments (*p* < 0.05) according to Tukey’s post hoc test. LoQ: limit of quantification.

The absolute abundances of the genes were obtained from ddPCR for each treatment (Fig. 2). The abundance of *sul1* was significantly lower in the mineral fertilization (NPK) and unamended treatments than that in the biochar soil treatment. Similarly, the abundance of *sul2* was lower in the anaerobic digestion, NPK, and unamended treatments than in the untreated compost and biochar soil treatments. Moreover, untreated compost, NPK, and unamended treatments showed lower *tetA* abundance than those in anaerobic digestion, biochar compost, and biochar soil treatments (the difference was not statistically significant between untreated compost and biochar compost). Regarding the MGE genes, anaerobic digestion, biochar compost, NPK, and unamended treatments presented a lower *intl1* gene abundance than that in the untreated compost. Finally, the abundance of *tnpA-04* was lower in the unamended treatment than in the untreated compost treatment.

The most abundant gene in the studied soils was *tnpA-04* in all treatments, followed by *sul1* in the biochar compost, biochar soil, and untreated compost treatments; *sul2* in the NPK and unamended treatments; and *tetA* in the anaerobic digestion treatment. In contrast, the least abundant gene in almost all treatments was *tetX*, with values were under the limit of quantification, with the NPK treatment being an exception in which the least abundant gene was *sul1*.

**Figure 3:**
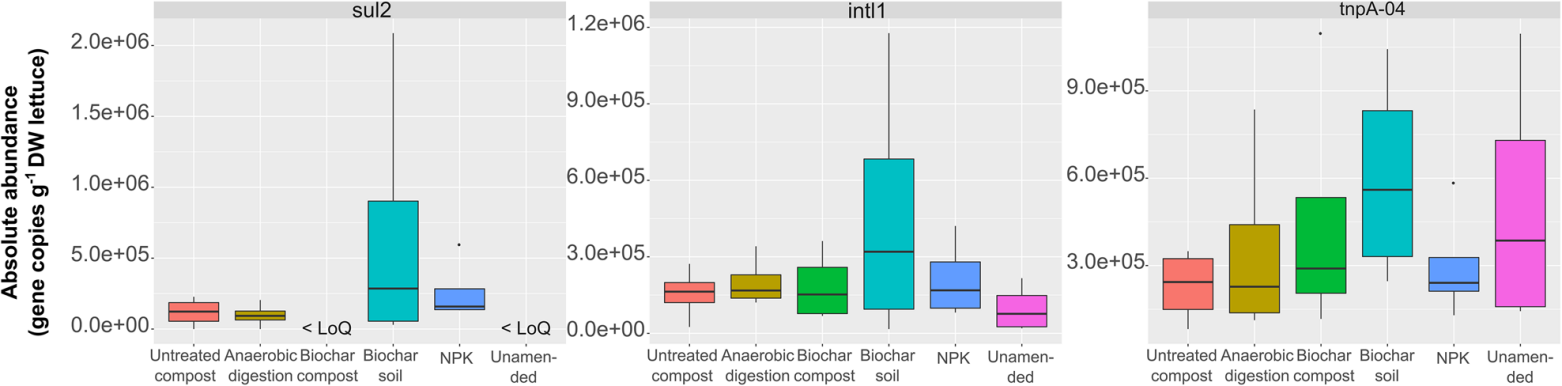
Absolute abundances of antibiotic resistance genes (ARGs) and mobile genetic element (MGE) genes in the lettuce samples according to the ddPCR analysis, expressed as gene copies per g of DW lettuce (n = 4). No statistically significant differences were found among the treatments. LoQ: limit of quantification.

With respect to lettuce plants, no statistically significant differences were found among the treatments in terms of ARG and MGE gene absolute abundances (Fig. 3). The most abundant gene in all treatments (expressed as gene copies 10^5^ g^−1^ DW lettuce) was *tnpA-04* (2.3 to 6.0, in untreated compost and biochar soil treatments, respectively), except in the biochar soil treatment, in which *sul2* was most abundant (6.7). In contrast, the abundances of *sul1*, *tetA*, and *tetX* were below the quantification limit.

The results of HT-qPCR showed that out of the 76 ARGs and 17 MGE genes targeted in the soils, 69 and 17 genes were amplified, respectively. Out of the 16 ARGs and five MGE genes targeted in lettuce plants, eight and three genes were amplified, respectively. No statistically significant differences in gene families were detected between the treatments in any soil (Supplementary Fig. S1) or lettuce plant (Supplementary Fig. S2). Regarding individual genes, we just found a higher relative abundance of a gene conferring resistance to aminoglycosides (*aph3-III*) in soils amended with untreated compost than in the rest of the treatments. Moreover, we compared the relative abundance of individual genes in both compartments (i.e., soil and lettuce). We found significant differences in 16 of the 21 common genes studied between soils and lettuce (Supplementary Table S4). In all cases, the relative abundance of the genes was higher in soils than in lettuce.

**Figure 4:**
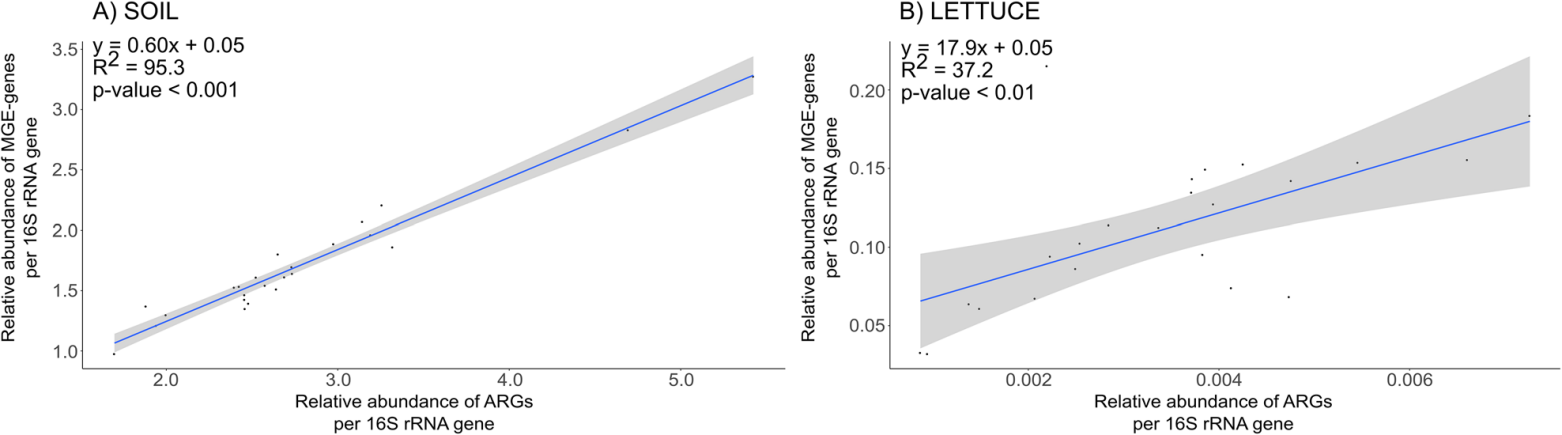
Results of the ordinary least squares (OLS) regression analysis of the relationship between the relative abundance of antibiotic resistance genes (ARGs) and mobile genetic element (MGE) genes in (**A**) soils and (**B**) lettuce plants. The line represents the best-fit curve. The shaded area represents the 95% confidence interval for the fitted OLS regression.

Furthermore, the ordinary least squares (OLS) regression model showed that the relative abundance of MGE genes was linearly and positively correlated with the relative abundance of ARGs in the studied soils (R^2^ = 95.3, *p* < 0.001) (Fig. 4A) and lettuce plants (R^2^ = 37.2, *p* < 0.01) (Fig. 4B).

### Soil bacterial community composition

Regarding the soil bacterial community composition, 99%, 93%, 84%, 47% and 6% of the reads were taxonomically classified at the class, order, family, genus, and species levels, respectively. The two most abundant classes were Alphaproteobacteria and Verrucomicrobiae, accounting for more than 60% of the relative abundance of bacteria; the exception was the anaerobic digestion treatment, in which the second most abundant class was Bacilli (Supplementary Fig. S3). The values of the Shannon’s diversity index (H’) ranged from 3.87 (in both the unamended and biochar compost treatments) to 4.12 (in the NPK treatment). No statistically significant differences were found among the treatments in terms of (i) taxa at class, order or genus levels; (ii) H’ index; or (iii) composition according to the ANOSIM test. Furthermore, no clustering of treatments was observed in the principal coordinates analysis (Supplementary Fig. S4).

## Discussion

Organic amendments of animal origin are important sources of antibiotic residues and microflora containing resistance genes (ARGs) in their genome^29^. Their dissemination in the environment and the associated risks to human and animal health are complex problems that require urgent action. Even though composting offers an environmentally friendly approach to biodegradation of antibiotic residues or ARGs to some extent^30^, some antibiotic residues and ARGs remain in the organic amendments. Therefore, it is essential to develop effective treatment methods for organic amendments of animal origin to reduce the risk.

The effectiveness of different management processes may differ depending on the type of organic amendment and whether it is in a solid, liquid, or semi-liquid state. In the present study, the best management processes were specific to each organic amendment. In general, the success was lower in compost than in the other two amendments, probably because it was more difficult for the treatment effect to spread well in this solid matrix.

Previous research suggested that anaerobic digestion may be able to remove pathogens and ARGs, with thermophilic digestion being particularly effective^31,32^. In a former study, the thermophilic digestion of dairy manure reduced the relative abundance of *intl1*, *sul1*, *sul2*, and *tetX*^32^. In contrast, an increase in *sul1*, *sul2*^33^, and *tetA*^34^ was reported as a result of thermophilic digestion of cattle and pig manure, respectively. These inconsistent results may be attributable to the different characteristics of the raw materials, digestion parameters, and bacterial community composition, among others. The increase of *tet* gene abundances at AD higher temperatures could be associated with the specific groups of bacteria harboring them and their capacity to grow under these environmental conditions^35^. Also, some *tet* genes (i.e., *tetX, tetW*) were considered genes with high dissemination potential in previous studies due to their association with MGEs^36,37^, and under such conditions they could be transferred among a wide range of bacteria. Remarkably, the impact of anaerobic processes on ARG reduction could be gene-specific^38^. In the present study, anaerobic digestion was among the best treatments for the three amendments. Surprisingly, the success rate was lower at higher digestion temperatures.

Similarly, the application of biochar to the three amendments (slurry, manure, and compost) was a promising treatment. A previous study^39^ evaluated the effects of applying rice straw biochar and mushroom biochar during chicken manure composting on ARG removal (including *sul1*, *sul2*, *tetA*, and *tetX* genes). The removal rate of *sul1*, *sul2*, *tetA* and *intl1* genes by adding biochar 5% was significantly higher than the control treatment during composting^40^. At the end of the composting process, the addition of biochar led to a decrease of the relative abundance of Bacteroidetes and Firmicutes^40^. Biochar application may potentially alter the resistome profile as a consequence of the changes in bacterial community composition, which is the primary determinant of ARG content^41^. Biochar can also adsorb heavy metals and reduce their bioavailability, thus reducing the selective pressure on ARB^42^.

Nanoparticles disrupt intracellular metabolism, damage DNA, and inhibit cell proliferation^43^. They can also enhance HGT via oxidative stress and increase the expression of mating pair formation^44^. In the present study, the application of nZVI 2% had the greatest success in removing all tested genes, but the removal rate was negative for almost every gene in the case of the compost. Another study also reported an increase in the relative abundance of *intl1*, *sul2*, and *tetC* in composted chicken manure 21 d after the application of 100 and 600 mg kg^−1^ of nZVI^45^. However, 42 d after the nZVI application, the relative abundance of the abovementioned genes decreased. This may have happened because nanoparticles reduced bacterial biomass and induced a succession of the bacterial community in the late stages of composting^45^.

In the present study, regarding ozonation, the abundances of ARGs and MGE genes increased in general compared to those in the control treatments. These results can be attributed to the consumption of ozone by organic constituents present in the amendments, such as humic substances, carbohydrates, and fatty acids^46^. Moreover, ozone damages the cell surface before releasing DNA, and intact ARGs may be transferred through HGT^47^. In another study, the inactivation of ARGs increased in municipal wastewater when the ozone concentration increased from 27 to 61 mg L^−1^^48^.

Finally, the enrichment of ARGs and MGE genes observed in both manure and compost after SMS application (observed only with the 1:1 dose in manure) might be attributable to (i) the nutrients provided by the SMS, which led to the proliferation of bacterial hosts carrying ARGs and MGEs in the amendment or (ii) SMS-derived ARG and MGE survival and transfer to both manure and compost.

The results of the microcosm experiment with lettuce plants differed depending on the specific genes and the method used to quantify them. The risk tendency discrepancy detected between ddPCR and HT-qPCR may be attributable to the reaction volume (21 µL vs. 100 nL in ddPCR vs. HT-qPCR, respectively), primer sequences employed, annealing temperature (specific vs. general), and abundance units (absolute vs. relative). Droplet digital PCR generates up to 20,000 droplets and a PCR reaction occurs in each droplet. In terms of sensitivity, the lower limit of detection of ddPCR was 10 times more efficient than that of qPCR^49^. Therefore, we believe that HT-qPCR is useful for quantifying a wide range of genes simultaneously, but the results obtained should be confirmed with another method, such as ddPCR. In our case, we chose to study tetracyclines and sulfonamides in detail because they accounted for 31% and 8% of the total antimicrobial sales for use in food-producing animals in 31 European countries in 2018, respectively^50^. Although sulfonamides are used less frequently than tetracyclines, the main sulfonamide resistance genes (*sul1* and *sul2*) are often located on transposable elements of self-transferable or mobilizable broad-host-range plasmids and occur in a wide range of bacterial species^51^. Furthermore, both antibiotics were categorized as highly important antimicrobials for human medicine by the World Health Organization^52^. Tetracycline and sulfonamide resistance genes have frequently been reported in manure and soil^53,54^.

In terms of the results obtained by ddPCR in the studied soils, anaerobic digestion led to a 38% and 83% reduction in *sul2* and *intl1* absolute abundance, respectively, compared to those in the untreated compost. In another study, dairy manure application resulted in higher abundances of *intl1* and *sul1* in soils fertilized with slurry digestate and untreated slurry than those in mineral fertilization and unamended soils^55^. The application of biochar to compost also led to a 60% reduction in *intl1* gene abundance compared with that in the untreated compost. In another study, the application of 0.5% biochar to soil decreased the ARG abundance in unplanted soil, but failed to remove ARGs from soil planted with *Brassica chinensis* L.^56^. As expected, in general, we found that the application of compost increased the soil antimicrobial resistance and mobile element genes, as both mineral fertilization and unamended soil treatments presented the lowest ARG and MGE abundances.

Fortunately, we detected lower abundances of ARGs and MGE genes in lettuce plants than in soil using HT-qPCR. Moreover, *sul1*, *tetA*, and *tetX* gene abundances were below the quantification limit in all treatments of lettuce plants according to ddPCR. These results were consistent with the findings of previous studies indicating that the resistome and mobilome are more diverse and abundant in rhizosphere soil than in plants after the application of organic amendments^36,56,57^. Furthermore, ordinary least squares regression models pointed to the key role of MGEs in shaping the pattern of ARGs.

No differences were found between treatments in terms of soil bacterial diversity and composition. Therefore, we can argue that the changes in the antimicrobial resistance and mobile element genes were not a consequence of the modification of the bacterial communities.

In summary, anaerobic digestion and biochar addition could be beneficial for reducing the risk of antimicrobial resistance and mobile element genes in organic amendments of animal origin before they are applied to soils. However, further confirmatory studies are necessary before applying these methods on a large scale. Ideally, the treatment or a combination of treatments should be inexpensive and feasible for application on livestock farms.

## Materials and methods

### Preliminary experiment

The amendments used in this study were provided by a dairy cow farm located in Basque Country (Spain). Three types of amendment (slurry, manure, and compost) were used. The slurry was collected from the outlet of a slurry pond, manure was collected from cow beddings (made from feces, urine, and wheat straw), and compost was sampled from manure piles that had been stored for six months. Both the manure and compost samples were collected and placed in polyethylene bags, whereas the slurry sample was placed in a plastic barrel. The samples were immediately transferred to the laboratory and stored at 4 °C until further use. The physicochemical properties of the amendments are presented in Supplementary Table S1.

The following 11 management processes were tested in the preliminary experiment: anaerobic digestion at 55 °C (AD 55 °C), 75 °C (AD 75 °C), and 90 °C (AD 90 °C); biochar addition at the rate of 0.5% (biochar 0.5%), 2% (biochar 2%), and 5% dry weight (DW; biochar 5%); zerovalent iron nanoparticle addition (nZVI) at 1% (nZVI 1%) and 2% DW (nZVI 2%); ozonation at 2 ppm (ozonation), and spent mushroom substrate addition (SMS) at 1:0.5 (SMS 1:0.5) and 1:1 w:w (SMS 1:1).

For anaerobic digestion, 16, 23, and 80 g FW of compost, manure, and slurry, respectively, were placed in syringes, and distilled water was added to make a final volume of 80 mL. These samples were incubated at the corresponding temperatures in an incubator for 21 d. The rest of the management was carried out at room temperature in darkness. First, 100 g FW of each amendment were placed in plastic pots. Biochar was manually added to the amendments and thoroughly mixed. It had the following physicochemical properties: DW = 53%, pH = 7.5, organic matter (OM) = 69%, electrical conductivity (EC) = 4.1 mS cm^−1^, total N = 0.7%, K = 13 g kg^−1^, P = 2.2 g kg^−1^, and metal concentrations = 0.56, 18, 23, 14, and 103 mg kg^−1^ for Cd, Cr, Cu, Ni, and Zn, respectively. Zerovalent iron nanoparticles (Nanofer 25S, aqueous dispersion of Fe(0) nanoparticles, NANO IRON s.r.o., Czech Republici) were added to the amendments in the form of slurry and thoroughly mixed; nZVI were applied twice, one week apart. For ozonation, the amendments were placed on a tray and subjected to ozone exposure in an ozonation chamber (2 ppm) for three weeks. Finally, the SMS obtained from the Mushroom Research Technological Center of La Rioja (Spain) was mixed with the amendments. The SMS had the following physicochemical properties: pH = 4.65, OM = 71%, total N = 0.49%, K = 4.3 g kg^−1^, and P = 0.62 g kg^−1^. All the management processes had their respective untreated controls (n = 1), which were under the same experimental conditions. For every treatment, two DNA extractions were performed at the beginning (day 0) and at the end of the process (day 21).

### Microcosm experiment

Before the beginning of the microcosm experiment, the following physicochemical properties were again determined in the compost, according to standard methods^58^: DW = 24%, pH = 9.1, C/N ratio = 14.6, EC = 0.54 mS cm^−1^, and metal concentrations = 0.49, 29, 229, 19, 9.4, 868 mg kg^-1^ DW for Cd, Cr, Cu, Ni, Pb, and Zn, respectively. The experimental soil was collected from the upper 30 cm layer of a semi-natural grassland field, which, to our knowledge, has never been amended. After collection, the soil was sieved to < 4 mm particles and was filled in 2 kg pots. The soil had the following physicochemical properties: clay loamy texture, OM = 5.4%, pH = 6.0, EC = 0.04 mS cm^−1^, total N = 0.22%, Olsen P = 2.3 mg kg^−1^ DW soil, and a K^+^ content of 97 mg kg^−1^ DW soil.

The following treatments were tested in the microcosm experiment: (i) untreated compost, (ii) compost subjected to anaerobic digestion at 75 °C for 21 d (anaerobic digestion), (iii) biochar 5% w/w to compost (biochar compost), (iv) biochar 5% w/w to soil (biochar soil), (v) mineral fertilization [NPK, N as NH_4_NO_3_ (33.5%), P as P_2_O_5_ (18%), and K as K_2_O (60%)], and (vi) unamended treatment. The compost dose was adjusted to provide an equivalent of 150 kg N ha^−1^ for lettuce plants. The compost was manually incorporated into the soil, thoroughly mixed (homogenized), and left to stabilize for two weeks. Lettuce seedlings were planted and bottom-watered every 2–3 days during the experimental period. The microcosm experiment was carried out in a growth chamber under the following controlled conditions: 14/10 h light/dark cycle, 20/16 °C day/night temperature, 70% relative humidity, and a photosynthetic photon flux density of 150 µmol photon m^−2^ s^−1^. Each treatment was replicated four times. After one month of growth, the plants were harvested and soil samples were collected.

### Quantification of antibiotic resistance genes and mobile genetic elements

Total DNA (i.e., both plasmid and genomic) was extracted from the amendments and soil samples (0.25 g DW) using the Qiagen DNeasy PowerSoil Pro Kit (Qiagen, Carlsbad, CA, USA) according to the manufacturer’s instructions, and from the plant samples using the innuPREP Plant DNA Kit (Analytik Jena, Jena, Germany). The quality of amendment, soil, and plant DNA was assessed using a NanoDrop™ One spectrophotometer (Thermo Scientific, Wilmington, DE, USA). The extracted DNA was stored at − 20 °C until further analysis.

Absolute quantification of ARGs and MGE genes in the preliminary and microcosm experiments was conducted using a droplet digital PCR (ddPCR, Bio-Rad Laboratories Inc., Hercules, CA, USA). Two sulfonamide (*sul1* and *sul2*) and two tetracycline (*tetA* and *tetX*) resistance genes, as well as two MGE genes (*intl1* and *tnpA-04*) and the 16S rRNA gene were analyzed (see primers and PCR conditions in Supplementary Table S2). Prior to generating droplets for ARGs and MGE genes, the DNA was digested with the XbaI restriction enzyme (Takara Bio, CA, USA) according to the manufacturer’s instructions. The reaction mixture (total volume of 25 µL) consisted of 12.5 µL of QX200 ddPCR EvaGreen Supermix (Bio-Rad), 0.50 µL each of forward and reverse primers (final concentration 10 nM each), 1.0 µL of the digested DNA extract (for 16S rRNA quantification, the DNA was diluted to 0.1 ng µL^−1^), and 10.5 Milli-Q water. The reaction mixture was added to a 96-well plate, sealed with foil, homogenized by vortexing, and centrifuged at 1000 g for 1 min. Aliquots of 21 µL ddPCR reaction mixture were dispensed into the sample well of the DG8 Droplet Generation Cartridge (Bio-Rad), and 70 µL of the QX200 Droplet Generation Oil for EvaGreen were added to the oil wells. Droplet generation was performed using a QX200 Droplet Generator (Bio-Rad). The generated droplets were transferred to a 96-well plate and incubated at 180 °C using a PX1 PCR plate sealer. The 96-well plate was then transferred to a C1000 Touch Thermal Cycler for PCR amplification. After thermal cycling, the plate was transferred to a QX200 droplet reader for data acquisition. Data analysis was performed using the QuantaSoft software (v.1.4., Bio-Rad). Target gene copies were quantified in duplicate, and positive and no-template controls were included in each ddPCR assay.

Furthermore, the relative abundance of ARGs and MGE genes in each sample of the microcosm experiment was analyzed using customized primer sets in a SmartChip qPCR system (TakaraBio) by Resistomap Oy (Helsinki, Finland). A total of 96 and 24 validated primer sets were used for soils and plants, respectively (see the list of primers in Supplementary Table S3); they were selected because they tested positive in a previous pre-screening analysis carried out with 384 genes. The quantified genes included (i) 76 and 16 primer sets (for soils and plants, respectively) targeting the ARGs conferring resistance against all major classes of antibiotics [aminoglycoside, β-lactam, FCA (phenicol, quinolone), MLSB (macrolide, lincosamide, streptogramin B), multidrug (i.e., those conferring resistance to more than one antibiotic), other (peptide, triclosan, mercury, hydrocarbon), sulfonamide, tetracycline, trimethoprim, and vancomycin]; (ii) 17 and 5 primer sets (for soils and plants, respectively) targeting MGE genes; and (iii) additional taxonomic reference genes (for Bacteroidetes, Firmicutes, and 16S rRNA).

PCR cycling conditions and initial data processing were the same as described previously^59–61^. A threshold cycle (C_T_) of 27 was used as the detection limit^53^. Detection of each ARG or MGE gene was considered positive when two out of three technical replicates for each sample were detected. The ${2}^{{-\mathrm{\Delta C}}_{T}}$ method was used to calculate the ARG and MGE gene relative abundances, normalized to the abundance of the 16S rRNA reference gene [where ${\mathrm{\Delta C}}_{T}= {C}_{T(target gene)}- {C}_{T(16S rRNA gene)}$^62^].

### Amplicon sequencing of soil bacterial communities

The prokaryotic 16S rRNA hypervariable region V4 was targeted using 519F (adapted from Ovreås et al.^63^) and 806R^64^ adapter-linked primer pairs as described by Lanzen et al.^65^. Although the sequencing of this single V-region is widely used for taxonomic classification purposes, the single target condition may lead to limitations in the estimation of diversity^66^. Pair-ended sequencing was performed using Illumina MiSeq at the Genomics Facility of SGIker (University of the Basque Country, Spain). Quality control of the reads was performed using FASTQC software^67^. PCR primers were removed from the sequences using Cutadapt^68^. The resulting FASTQ files were further analyzed via *QIIME2*^69^ as follows: sequences were imported into QIIME2 as *PairedEndSequencesWithQuality*, after which both reads were joined by the *qiime vsearch join-pairs *plugin. Then, low-quality reads were filtered using the *QIIME quality-filter Q-score-joined* command with the default options. In the next step, deblur^70^ was used for denoising (qiime deblur denoise-16S), after which the resulting reads were classified using the *qiime feature-classifier classify-sklearn* and *silva-132-99-nb-classifier.qza* command as the reference model for assigning a taxonomical classification to the amplicon sequence variants (ASVs). The singletons were removed by the *QIIME feature-table filter-features* command, and contaminant and unclassified ASVs were also removed by the *QIIME taxa filter-table*. Finally, a table summarizing the ASVs was created using a *qiime feature-table summarize* command*.*

### Statistical analysis

In the preliminary experiment, the removal rates were calculated following Yang et al.^71^ by first subtracting the abundances obtained on day 21 from those obtained on day 0 and then from those of the control treatment. Heatmaps were visualized with ggplot2^72^.

In the microcosm experiment, statistically significant differences among the treatments in both the absolute and relative abundances of ARGs and MGE genes (*p* < 0.05) for soil and lettuce were assessed with ANOVA and Tukey’s post-hoc tests using the agricolae R package^73^, followed by Bonferroni’s multiple comparisons test on the relative abundances of individual genes from HT-qPCR results. This same procedure was followed with the ASVs obtained from the amplicon sequencing. Ordinary least squares (OLS) regression models were used to assess the relationships between ARG and MGE gene relative abundances obtained by HT-qPCR analysis using R, and the results were visualized using the ggplot2 package^72^. The analysis of similarities (ANOSIM) test was used to compared the mean of ranked dissimilarities between treatments to the mean of ranked dissimilarities within treatments. Principal coordinates analysis (PCoA) was used to visualize differences between treatments regarding soil microbial community composition with the ape package^74^. Bray–Curtis dissimilarity based on the abundance classes was calculated using the vegan package^75^.

## Supplementary Information

Supplementary Information.

## Data Availability

All sequence data have been deposited in the European Nucleotide Archive under the study accession number PRJEB49515.
