# Supplementary material for: Organic amendment treatments for antimicrobial resistance and mobile element genes risk reduction in soil-crop systems
Source: Sci Rep. 2023 Jan 17;13:863. doi: 10.1038/s41598-023-27840-9 (PMC9845208; doi:10.1038/s41598-023-27840-9)
Supplement: Supplementary file 1 — Supplementary Information. [file 41598_2023_27840_MOESM1_ESM.pdf]

Organic amendment treatments for resistome risk reduction in soil-crop systems

Leire Jauregi*, Aitor González, Carlos Garbisu, Lur Epelde

Supplementary Table S1. Physicochemical properties of the organic amendments studied in the preliminary experiment.

Property	Compost	Manure	Slurry
**Dry matter (%)**	32.61	22.42	6.56
**Electrical conductivity (mS cm^{-1})**	1.03	1.08	1.00
**pH**	8.69	8.91	8.67
**Organic matter (% DW)**	66.23	86.74	78.27
**Organic C (%)**	12.56	11.31	2.99
**N (%)**	1.00	0.58	0.32
**C/N**	12.56	20.56	29.85
**K_2O (% DW)**	2.10	3.10	4.44
**P_2O_5 (% DW)**	0.59	0.70	1.16
**Cd (mg kg^{-1} DW)**	0.15	0.06	0.14
**Co (mg kg^{-1} DW)**	1.68	1.40	3.25
**Cr (mg kg^{-1} DW)**	7.06	6.74	7.08
**Cu (mg kg^{-1} DW)**	55.31	50.71	116.94
**Ni (mg kg^{-1} DW)**	10.63	4.61	10.78
**Pb (mg kg^{-1} DW)**	2.72	1.44	2.76
**Zn (mg kg^{-1}DW)**	203.07	193.97	441.4

Supplementary Table S2. Primers and PCR conditions for droplet digital PCR (ddPCR) analysis.

Gene	Primers	Amplicon size (bp)	Cycling conditions	Reference
**16S rRNA**	F: CCTACGGGAGGCAGCAG<br>R: ATTACCGCGGCTGCTGG	194	95 ºC for 5 min<br>40 cycles of 95 ºC for 30 s and 60 ºC for 1 min, with a ramping rate of 2.5 ºC s^{-1}<br>4 ºC for 5 min<br>90 ºC for 5 min	(1)
**tetA**	F: GCTACATCCTGCTTGCCTTC<br>R: CATAGATCGCCGTGAAGAGG	210	95 ºC for 5 min<br>40 cycles of 95 ºC for 30 s and 62.5 ºC for 1 min, with a ramping rate of 2.5 ºC s^{-1}<br>4 ºC for 5 min<br>90 ºC for 5 min	(2)
**tetX**	F: AGCCTTACCAATGGGTGTAAA<br>R: TTCTTACCTTGGACATCCCG	278	95 ºC for 5 min<br>40 cycles of 95 ºC for 30 s and 57.5 ºC for 1 min, with a ramping rate of 2.5 ºC s^{-1}<br>4 ºC for 5 min<br>90 ºC for 5 min	(3)
**sul1**	F: CCGTTGGCCTTCCTGTAAAG<br>R: TTGCCGATCGCGTGAAGT	67	95 ºC for 5 min<br>40 cycles of 95 ºC for 30 s and 60 ºC for 1 min, with a ramping rate of 2.5 ºC s^{-1}<br>4 ºC for 5 min<br>90 ºC for 5 min	(4)
**sul2**	F: CGGCTGCGCTTCGATT<br>R: CGCGCGCAGAAAGGATT	60	95 ºC for 5 min<br>40 cycles of 95 ºC for 30 s and 60 ºC for 1 min, with a ramping rate of 2.5 ºC s^{-1}<br>4 ºC for 5 min<br>90 ºC for 5 min	(5)
**intl1**	F: GCCTTGATGTTACCCGAGAG<br>R: GATCGGTCGAATGCGTGT	196	95 ºC for 5 min<br>40 cycles of 95 ºC for 30 s and 61 ºC for 1 min, with a ramping rate of 2.5 ºC s^{-1}<br>4 ºC for 5 min<br>90 ºC for 5 min	(6)
**tnpA-04**	F: CCGATCACGGAAAGCTCAAG<br>R: GGCTCGCATGACTTCGAATC	101	95 ºC for 5 min<br>40 cycles of 95 ºC for 30 s and 61 ºC for 1 min, with a ramping rate of 2.5 ºC s^{-1}<br>4 ºC for 5 min<br>90 ºC for 5 min	(7)

Supplementary Table S3. Primer sets used in high-throughput qPCR (HT-qPCR) analysis.

Gene	Forward Primer	Reverse Primer	Target antibiotics (major)
**16S rRNA**	GGGTTGCGCTCGTTGC	ATGGYGTCGTCAGCTCGTG	16S rRNA
**Bacteroidetes**	GGARCATGTGGTTTAATTCGATGAT	AGCTGACGACAACCATGCAG	Taxanomic
**Firmicutes**	GGAGYATGTGGTTTAATTCGAAGCA	AGCTGACGACAACCATGCAC	Taxanomic
**aac(3)-iid_iia**	CGATGGTCGCGGTTGGTC	TCGGCGTAGTGCAATGCG	Aminoglycoside
**aac(6)-ig**	GCGATGTTAGAAGCCTCAATTCG	CACACTTCGGCCTGTCGAA	Aminoglycoside
**aac(6)-ir**	GCTATAACGATCAGCAGCAAGC	CGCGATGCATGGCATGAC	Aminoglycoside
**aac(6)-is_iu_ix**	AAGCTTACTCTGGCCTGATCATG	TGCCTGAACGTCGATATTCAGG	Aminoglycoside
**aac(6')-Iy**	GCCTCAATCCGCCACGATTA	ACGCGCTCTGTTTCCTCAAA	Aminoglycoside
**aac3-IVa**	CCAACACGACGCTGCATC	GCTGTCGCCACAATGTCG	Aminoglycoside
**aac6-aph2**	CCAAGAGCAATAAGGGCATACCAA	GCCACACTATCATAACCACTACCG	Aminoglycoside
**aadA1**	TGTACGGCTCCGCAGTG	CACGGAATGATGTCGTCGTG	Aminoglycoside
**aadA16**	ACGGTGGCCTGAAGCC	GAATTGCAGTTCCCGTCTGG	Aminoglycoside
**aadA5**	ATCACGATCTTGCGATTTTGCT	CTGCGGATGGGCCTAGAAG	Aminoglycoside
**aadA6**	CCATCGAGCGTCATCTGGAA	CCCGTCTGGCCGGATAAC	Aminoglycoside
**aadA7**	CACTCCGCGCCTTGGA	TGTGGCGGGCTCGAAG	Aminoglycoside
**aadB**	CCTGCTTGGTGGGCAGAC	CGGCACGCAAGACCTCAA	Aminoglycoside
**aph3-III**	CAGAAGGCAATGTCATACCACTTG	GACAGCCGCTTAGCCGAA	Aminoglycoside
**aph4-Ib**	GGGAACACCGTGCTCACC	GTTGGTCCCGTGCAGGTC	Aminoglycoside
**aph6-Ia**	CGCTGGGAGCTGAAGAGG	AGCATCGTGCTGCTCTCC	Aminoglycoside
**apmA**	GGCGCACATGCATTCATCA	CTATACTCCAGTCCCACCATTTGA	Aminoglycoside
**armA**	TCTTCGACGAATGAAAGAGTCG	GCTAATGGATTGAAGCCACAACC	Aminoglycoside
**spcN**	GCTATGTGCTGGTGGACTGG	GGAACCACTCGACGAACTCG	Aminoglycoside
**strB**	GCTCGGTCGTGAGAACAATCT	CAATTTCGGTCGCCTGGTAGT	Aminoglycoside
**bla1**	GCAAGTTGAAGCGAAAGAAAAGA	TACCAGTATCAATCGCATATACACCTAA	β-lactam
**blaACT**	AAGCCGCTCAAGCTGGA	GCCATATCCTGCACGTTGG	β-lactam
**blaBEL-nonmobile**	ATGTCCATGGCACAGACTGTG	CCTGTCTTGTCACCCGTTACC	β-lactam
**blaMIR**	CGGTCTGCCGTTACAGGTG	AAAGACCCGCGTCGTCATG	β-lactam
**blaOXY_1**	CGTTCAGGCGGCAGGTT	GCCGCGATATAAGATTTGAGAATT	β-lactam
**blaOXY_2**	AAAGGTGACCGCATTCGC	CCAGCGTCAGCTTGCG	β-lactam
**blaSFO**	CCGCCGCCATCCAGTA	GGGCCGCCAAGATGCT	β-lactam
**cphA**	GCGAGCTGCACAAGCTGAT	CGGCCCAGTCGCTCTTC	β-lactam
**intI1_1**	CGAACGAGTGGCGGAGGGTG	TACCCGAGAGCTTGGCACCCA	Integrons
**intI1_2**	CGAAGTCGAGGCATTTCTGTC	GCCTTCCAGAAAACCGAGGA	Integrons
**intl3**	CAGGTGCTGGGCATGGA	CCTGGGCAGCATCACCA	Integrons
**acrA**	GGTCTATCACCCTACGCGCTATC	GCGCGCACGAACATACC	MDR
**cefa_qacelta**	TAGTTGGCGAAGTAATCGCAAC	TGCGATGCCATAACCGATTATG	MDR
**czcA**	GCCTTGTTCATCGGCGAAC	GGCAATGTCGCCTTCGTTC	MDR
**emrD**	CTCAGCAGTATGGTGGTAAGCATT	ACCAGGCGCCGAAGAAC	MDR

**mepA**	ATCGGTCGCTCTTCGTTCAC	ATAAATAGGATCGAGCTGCTGGAT	MDR
**oprD**	ATGAAGTGGAGCGCCATTG	GGCCACGGCGAACTGA	MDR
**pbrT**	GATGCGCACTGGGCTTG	TCGGAATATGCGGAAATGCG	MDR
**sugE**	CTTAGTTATTGCTGGTCTGCTGGA	GCATCGGGTTAGCGGACTC	MDR
**ttgA**	ACGCCAATGCCAAACGATT	GTCACGGCGCAGCTTGA	MDR
**IncN_rep**	AGTTCACCACCTACTCGCTCCG	CAAGTTCTTCTGTTGGGATTCCG	MGE
**IS1111**	GTCTTAAGGTGGGCTGCGTG	CCCCGAATCTCATTGATCAGC	MGE
**IS1133**	GCAGCGTCGGGTTGGA	ACGCGTTCGAACAACTGTAATG	MGE
**IS1247_1**	CGGCCGTCACTGACCAA	TCGGCAGGTTGGTGACG	MGE
**IS1247_2**	TGGATCGACCGGTTCCAT	GCTGACCGAGCTGTCCATGT	MGE
**IS6100**	CGCACCGGCTTGATCAGTA	CTGCCACGCTCAATACCGA	MGE
**IS630**	CCGCCACCAGTGTGATGG	TTGGCGCTGACTGGATGC	MGE
**ISEcp1**	CATGCTCTGCGGTCACTTC	GACGCACCTTCTTGATGACC	MGE
**ISPps**	CACACTGCAAAAACGCATCCT	TGTCTTTGGCGTCACAGTTCTC	MGE
**orf37-IS26**	GCCGGGTTGTGCAAATAGAC	TGGCAATCTGTCGCTGCTG	MGE
**Tn5403**	AAGCGAATGGCGCGAAC	CGCGCAGGGTAAACTGC	MGE
**tnpA_2**	CCGATCACGGAAAGCTCAAG	GGCTCGCATGACTTCGAATC	MGE
**tnpA_3**	GGGCGGGTCGATTGAAA	GTGGGCGGGATCTGCTT	MGE
**trbC**	CGGYATWCCGSCSACRCTGCG	GCCACCTGYSBGCAGTCMCC	MGE
**ereA**	GATAATTCTGCTGGCGCACA	GCAGGCGTGGTCACAAC	MLSB
**erm35**	CCTTCAGTCAGAACCGGCAA	GCTGATTTGACAGTTGGTGGTG	MLSB
**erm36**	GGCGGACCGACTTGCAT	TCTGCGTTGACGACGGTTAC	MLSB
**ermE**	GTCACGCAGCTGGAGTTCG	CGGTGAAGCACAGCTCGAC	MLSB
**ermO**	GAGTACGCCCGCAAACG	GCGTTCGATCCGGAGGA	MLSB
**ermX**	TGATGACGGCTCAGTGG	GTGCACCAGCGCCTGA	MLSB
**lnuB**	GGATCGTTTACCAAAGGAGAAGG	AGCATAGCCTTCGTATCAGGAA	MLSB
**lnuC**	GGGTGTAGATGCTCTTCTTGGA	CTTTACCCGAAAGAGTTTCTACCG	MLSB
**mefA_1**	TAATTATCGCAGCAGCTGGTTC	GTTCCCAAACGGAGTATAAGAGTG	MLSB
**mefA_2**	CCGTAGCATTGGAACAGCTTTT	AAACGGAGTATAAGAGTGCTGCAA	MLSB
**mphA**	TCAGCGGGATGATCGACTG	GAGGGCGTAGAGGGCGTA	MLSB
**oleC**	CCCGGAGTCGATGTTCGA	GCCGAAGACGTACACGAACAG	MLSB
**pncA**	GCAATCGAGGCGGTGTTC	TTGCCGCAGCCAATTCA	MLSB
**vat(A)**	ATGAACGGAGCGAATCATCGG	CCATACCGATCCAAACGTCATTTC	MLSB
**bacA**	ATCCGCGGCACCCTGA	CCTGCTTGATGGACTTGATGAAGA	Other
**fabK**	CAGGAGCAGGAAATCCAAGC	CCAGCTTCCATTCCTTCTGC	Other
**mcr1**	CACATCGACGGCGTATTCTG	CAACGAGCATACCGACATCG	Other
**merA**	GTGCCGTCCAAGATCATG	GGTGGAAGTCCAGTAGGGTGA	Other
**ttgB**	TCGCCCTGGATGTACACCTT	ACCATTGCCGACATCAACAAC	Other
**catQ**	AGGTGCACTTACAGTATGACTGC	AACGTGGGAAGTTCTCGTCATAC	Phenicol
**cmlV**	GCCCTCATCACCGTCTTCG	GGACGTTGGCGATGGAGAG	Phenicol
**optrA**	GGTGGATGAAGTCCGTACGG	AGGTTAGACCTCCAAGAGCCA	Phenicol
**qepA**	GGGCATCGCGCTGTTC	GCGCATCGGTGAAGCC	Quinolone
**qnrB**	GCGACGTTCAGTGGTTCAGA	GCTGCTCGCCAGTCGAA	Quinolone

**qnrB4**	TCACCACCCGCACCTG	GGATATCTAAATCGCCCAGTTCC	Quinolone
**sul3**	TCCGTTCAGCGAATTGGTGCAG	TTCGTTCACGCCTTACACCAGC	Sulfonamide
**tetD**	AATTGCACTGCCTGCATTGC	GACAGATTGCCAGCAGCAGA	Tetracycline
**tetG**	TCGCGTTCCTGCTTGCC	CCGCGAGCGACAAACCA	Tetracycline
**tetL**	ATGGTTGTAGTTGCGCGCTATAT	ATCGCTGGACCGACTCCTT	Tetracycline
**tetM**	GGAGCGATTACAGAATTAGGAAGC	TCCATATGTCCTGGCGTGTC	Tetracycline
**tetPA**	GGAAACCTTAGTTCAGTGACTTGG	CCCATTTAACCACGCACTGAA	Tetracycline
**tetPB**	TGGGCGACAGTAGGCTTAGAA	TGACCCTACTGAAACATTAGAAATATACCT	Tetracycline
**tetR**	CCGTCAATGCGCTGATGAC	GCCAATCCATCGACAATCACC	Tetracycline
**tetW**	ATGAACATTCCCACCGTTATCTTT	ATATCGGCGGAGAGCTTATCC	Tetracycline
**dfrA27**	GCCGCTCAGGATCGGTA	GTCGAGATATGTAGCGTGTCG	Trimethoprim
**vanA**	GGGCTGTGAGGTCGGTTG	TTCAGTACAATGCGGCCGTTA	Vancomycin
**vanB**	TTGTCGGCGAAGTGGATCA	AGCCTTTTTCCGGCTCGTT	Vancomycin
**vanHB**	GAGGTTTCCGAGGCGACAA	CTCTCGGCGGCAGTCGTAT	Vancomycin
**vanTC**	ACAGTTGCCGCTGGTGAAG	CGTGGCTGGTCGATCAAAA	Vancomycin

Supplementary Table S4. Comparison of the relative abundances of antibiotic resistance genes and mobile genetic element genes in soil (S) and lettuce (L) samples for each treatment, based on Tukey's post-hoc test. ns: not significant; *: p < 0.05; **: p < 0.01; ***: p < 0.001.

Gene	Untreated compost	Anaerobic digestion	Biochar compost	Biochar soil	NPK	Unamended
**aac(6)-Ig**	S > L ***	S > L **	S > L **	S > L ***	S > L ***	S > L *
**aac3-IVa**	S > L ***	S > L **	S > L *	S > L ***	S > L **	S > L **
**aadA5**	S > L *	ns	ns	ns	ns	S > L *
**blaBEL-nonmobile**	ns	ns	ns	ns	ns	ns
**cmlV**	S > L ***	S > L **	S > L **	S > L ***	S > L *	S > L ***
**czcA**	S > L ***	S > L **	S > L **	S > L ***	S > L ***	S > L **
**erm35**	S > L **	S > L *	S > L *	S > L **	S > L **	S > L **
**ermX**	S > L ***	S > L **	S > L ***	S > L ***	S > L ***	S > L **
**fabK**	S > L ***	S > L ***	S > L ***	S > L ***	S > L **	S > L *
**intI1_1**	S > L ***	S > L **	S > L ***	S > L ***	S > L ***	S > L **
**intI1_2**	ns	ns	ns	S > L *	ns	ns
**IS6100**	S > L ***	S > L **	S > L **	S > L ***	S > L ***	S > L *
**ISEcp1**	S > L ***	S > L *	S > L **	S > L **	S > L **	S > L **
**mefA**	S > L ***	S > L *	S > L **	S > L ***	S > L **	S > L *
**mepA**	S > L ***	S > L **	S > L ***	S > L **	S > L ***	S > L **
**optrA**	S > L ***	S > L *	S > L ***	S > L ***	S > L ***	S > L ***
**orf37-IS26**	S > L ***	S > L **	S > L ***	ns	S > L ***	S > L **
**spcN**	S > L ***	S > L ***	S > L **	S > L ***	S > L ***	S > L ***
**tetPA**	ns	ns	ns	ns	ns	ns
**ttgA**	S > L ***	S > L *	S > L ***	S > L ***	S > L ***	S > L **

Supplementary Figure S1. Relative abundance (via HT-qPCR analysis) of antibiotic resistance genes and mobile genetic element genes in the studied soils.

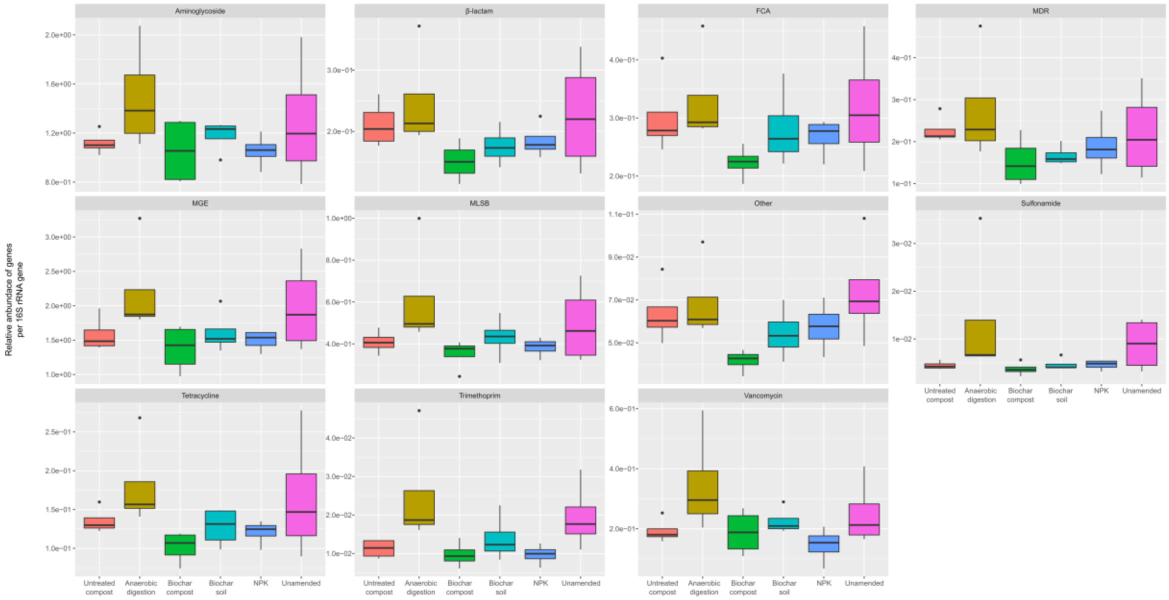

Supplementary Figure S2. Relative abundance (via HT-qPCR analysis) of antibiotic resistance genes and mobile genetic element genes in lettuce samples.

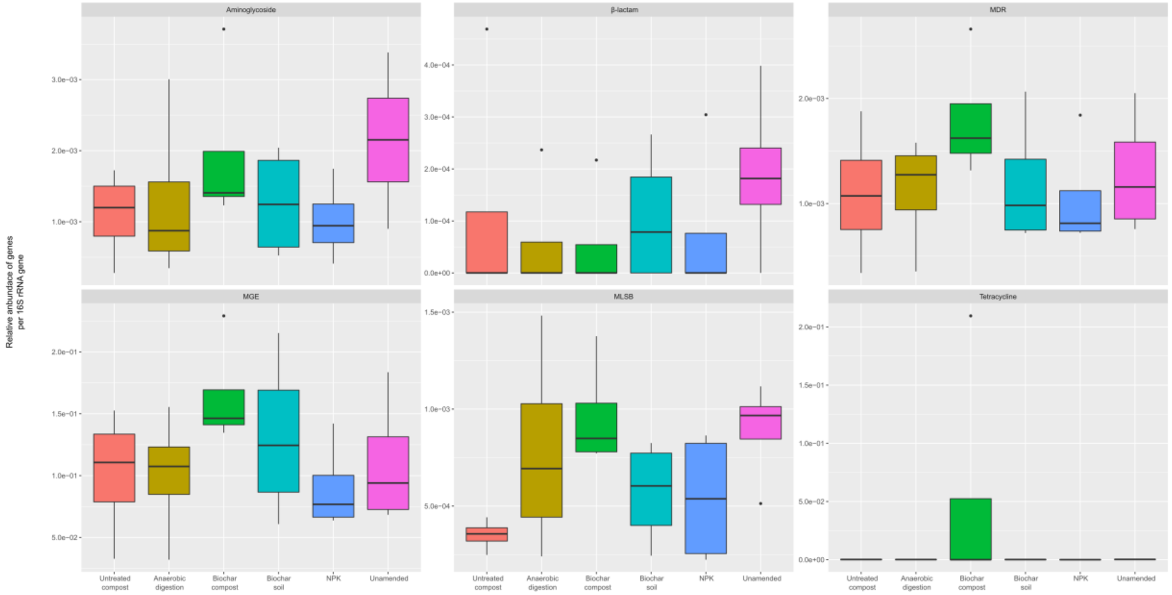

Supplementary Figure S3. The 30 most abundant bacterial classes in the studied soils.

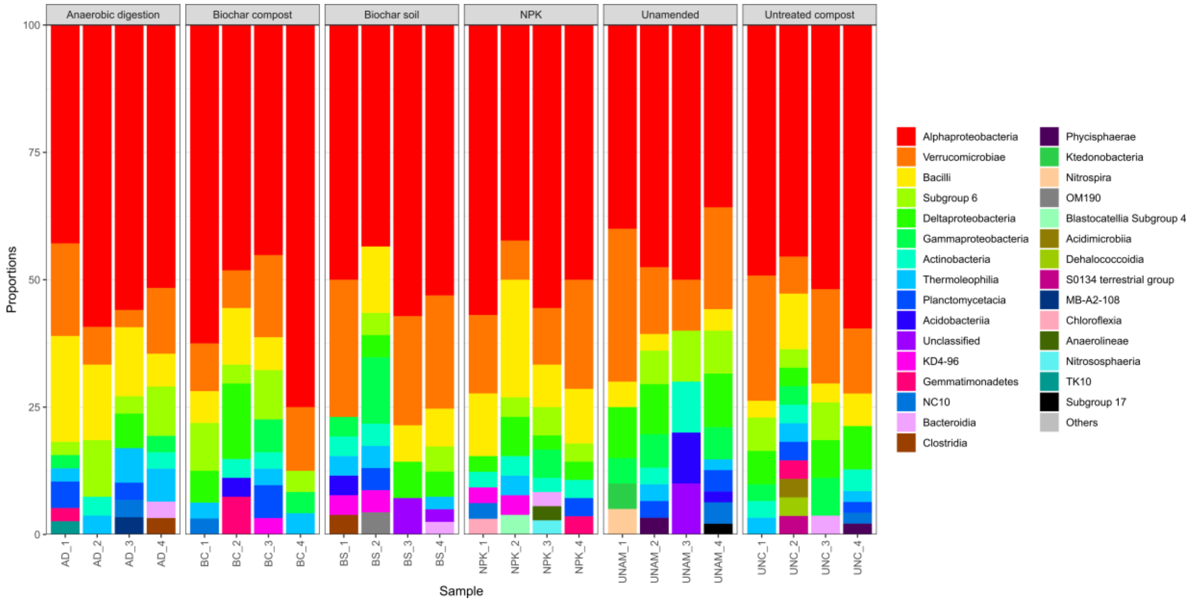

Supplementary Figure S4. Principal coordinates analysis (PCoA) plot of soil microbial community composition based on Bray-Curtis distance metric for bacterial class in the studied soils.

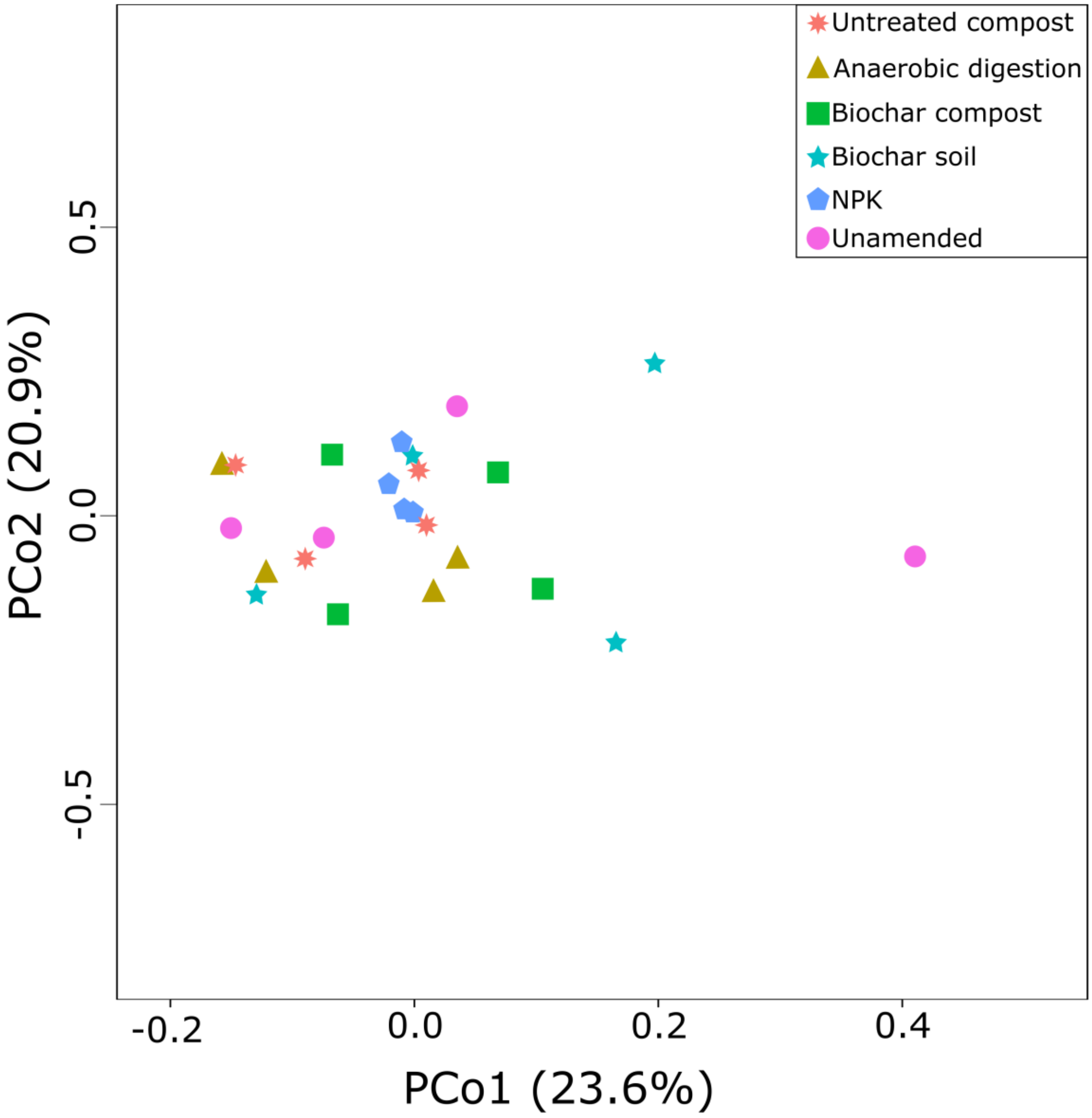

References

1. Muyzer, G., de Waal, E. C. & Uitterlinden, A. G. Profiling of complex microbial populations by denaturing gradient gel electrophoresis analysis of polymerase chain reaction-amplified genes coding for 16S rRNA. *Appl. Environ. Microbiol.* **59**, 695–700 (1993).
2. Ng, L. K., Martin, I., Alfa, M. & Mulvey, M. Multiplex PCR for the detection of tetracycline resistant genes. *Mol. Cell Probes* **15**, 209–215 (2001).
3. Diehl, D. L. & Lapara, T. M. Effect of temperature on the fate of genes encoding tetracycline resistance and the integrase of class 1 integrons within anaerobic and aerobic digesters treating municipal wastewater solids. *Environ. Sci. Techn.* **44**, 9128–9133 (2010).
4. Heuer, H. & Smalla, K. Manure and sulfadiazine synergistically increased bacterial antibiotic resistance in soil over at least two months. *Environ. Microbiol.* **9**, 657–666 (2007).
5. Heuer, H. *et al.* Fate of sulfadiazine administered to pigs and its quantitative effect on the dynamics of bacterial resistance genes in manure and manured soil. *Soil Biol. Biochem.* **40**, 1892–1900 (2008).
6. Barraud, O., Baclet, M. C., Denis, F. & Ploy, M. C. Quantitative multiplex real-time PCR for detecting class 1, 2 and 3 integrons. *J. Antimicrob. Chemother.* **65**,1642–1645 (2010).
7. Zhu, Y.G. *et al.* Diverse and abundant antibiotic resistance genes in Chinese swine farms. *P. Natl. Acad. Sci.* **110**, 3435–3440 (2013).
